# Identification of the Novel Gene Markers Based on the Gene Profile among Different Severity of Obstructive Sleep Apnea

**DOI:** 10.1155/2022/6517965

**Published:** 2022-10-04

**Authors:** Yi Ren, Yanyan Li, Xuemei Sui, Jinhe Yuan, Jun Lan, Xiayu Li, Yue Deng, Zhiping Xu, Xiu Cheng, Changjing Zhao, Junyu Lu

**Affiliations:** Department of Respiratory and Critical Care Medicine, The Fifth People's Hospital of Chongqing, Chongqing 400062, China

## Abstract

Obstructive sleep apnea (OSA) is caused by repeated blockage of the upper respiratory airways during sleep. The traditional evaluation methods for OSA severity are yet limited. This study aimed to screen gene signatures to effectively evaluate OSA severity. Expression profiles of peripheral blood mononuclear cells in the different severities of OSA patients were accessed from Gene Expression Omnibus (GEO) database. A total of 446 differentially expressed genes (DEGs) were screened among the varying severities of OSA samples by analysis of variance (ANOVA) test. A total of 1,152 DEGs were screened between the pre- and post-treatment OSA samples by using *t* test. Overlap of the two groups of DEGs was selected (88 DEGs) for Metascape enrichment analysis. Afterwards, Mfuzz package was used to perform soft clustering analysis on these 88 genes, by which 6 clusters were obtained. It was observed that the gene expression condition of the cluster 3 was positively associated with OSA severity degree; also, the gene expression condition in cluster 4 was negatively correlated with OSA severity. A total of 10 gene markers related to OSA progression were selected from cluster 3 and cluster 4. Their expression levels and correlation were analyzed. The marker genes in cluster 3 and cluster 4 were examined, finding that most genes were significantly correlated with apnea hypopnea index (AHI). An accurate and objective assessment of the severity of OSA is of great significance for formulating follow-up treatment strategies for patients with OSA. In this paper, a set of marker genes that can detect the severity of OSA were screened by bioinformatics methods, which could be jointly used with the traditional OSA diagnostic index to achieve a more reliable OSA severity evaluation.

## 1. Introduction

Obstructive sleep apnea (OSA) is a common and easily overlooked sleep breathing disorder caused by the upper respiratory tract collapse during sleep. Its clinical symptoms are loud snoring, daytime hypersomnia, and fatigue [1]. Therapeutic strategy largely depends on the severity of OSA, which is evaluated by apnea hypopnea index (AHI). AHI is defined as the times of episodes of apnea and hypopnea per hour during sleep [2]. According to evaluation criteria for severity of OSA issued by American Academy of Sleep Medicine (AASM; https://aasm.org/), severity of OSA is mild when AHI ranges from 5 to 15, moderate as AHI ranges from 15 to 30, and severe as AHI is larger than 30 [3]. Relevant studies show that AHI has certain limitations in assessing the severity of disease, which may be affected by factors such as gender and age [4], and the complications such as hypoxia caused by OSA cannot be fully reflected by the level of AHI [5]. Although AHI is still the gold standard for diagnosing the severity of OSA, it may not be comprehensive to evaluate the severity of OSA by using AHI alone. Therefore, it is urgent to search for powerful gene markers to assist in jointly examining the severity of OSA.

With the development of RNA sequencing, gene chip technology, and microarray tools, there is a growing body of literature that studies OSA-related genes, which provides several theoretic evidence for diagnosis, evaluation, and treatment of OSA [6]. For instance, Kun Li et al. [7] predicted OSA-related miRNAs and mRNA by a series of microarray analyses in 2017. Yuanyuan Cao et al. [8] screened and identified potential biomarkers of OSA and its related diseases by microarray analysis. Peng Lu et al. [9] established and validated the prognostic model of OSA based on microarray datasets and relevant bioinformatics analysis, providing a basis for further research on the molecular mechanism and potential targets of lipid metabolism regulation of OSA. However, few studies have involved gene markers related to different severity of OSA. Therefore, this study aimed to mine gene markers among the different severity OSA via microarray analysis.

This study downloaded the expression profile data of peripheral blood mononuclear cells in the patients with different severity OSA from public databases. Subsequently, differential expression analysis was performed to obtain DEGs. Functional enrichment analysis and soft clustering analysis were conducted to investigate the biological functions related to the DEGs and the expression states among different OSA severity, respectively. Finally, the correlation between marker genes expression and AHI was analyzed. Taken together, this study accessed expression profile data in different severity of OSA from GEO public database and screened relevant gene markers via a series of bioinformatics analysis, which could be jointly used with the traditional OSA diagnostic index to achieve a more reliable OSA severity evaluation and is of great significance for formulating appropriate treatment strategies for OSA patients.

## 2. Methods

### 2.1. Data Acquisition and Microarray Analysis

**Figure 1:**
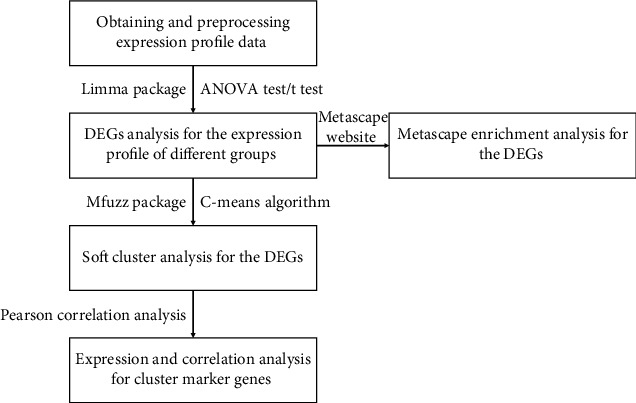
Flow chart of microarray analysis.

OSA-related expression matrix (GSE75097) was downloaded from Gene Expression Omnibus (GEO) database (https://www.ncbi.nlm.nih.gov/geo/) [10]. The severity of OSA was graded according to the boundary value of AHI. The dataset in this study included 6 primary snoring (PS) (AHI = 5 − 15), 16 moderate to severe OSA (MSO) (AHI = 15 − 30), 12 very severe OSA (VSO) (AHI > 30), and 14 very severe OSA with long-term continuous positive airway pressure treatment (VSOC) patients' whole genome expression profiles of peripheral blood mononuclear cells and related clinical data. Gene expression data were acquired by GPL 10904 detection platform. Based on the above dataset, we performed the following microarray analyses (Figure 1).

### 2.2. Data Preprocessing and Differential Expression Analysis

Limma package [11] was used to read the expression matrix. Background correction and quantile normalization were undertaken [12]. Next, probes that could not match gene symbol were removed. In the case of multiple probes corresponding to one gene, the probe with the highest average expression was retained to filter genes.

Significantly DEGs (*p* < 0.05) were determined from PS, MSO, and VSO groups using ANOVA test based on preprocessed expression profile data. Significantly DEGs (*p* < 0.05) were determined from VSO and VSOC groups by using *t* test. Lastly, the interaction of the above groups was taken as DEGs related to different severity of OSA.

### 2.3. Metascape Enrichment Analysis

To analyze the biological functions and signaling pathways involved in DEGs related to different OSA severity, we performed Metascape enrichment analysis. Metascape, a website tool (https://metascape.org/gp/index.html#/main/step1), integrates more than 40 microarray databases and can conduct multiple microarray analyses on gene lists. Metascape enrichment analysis can simplify redundant analysis results and analyze less correlated terms [13] and display the most important experimental results with concise bargraph. Moreover, enriched biological pathways can be expressed in a network manner, which is more conducive to understanding the relationship between pathways or biological processes.

### 2.4. Soft Clustering Analysis

Mfuzz package [14] (http://www.bioconductor.org/packages/release/bioc/html/Mfuzz.html, version 2.6.1) was applied in this study for soft clustering analysis on DEGs related to different OSA severity. Acore threshold value was set as 0.6. Mfuzz package applied fuzzy c-means algorithm. Several time-course experiments found that it is difficult to distinguish DEGs in different time courses by a clear boundary [15–17]. Therefore, these genes are more suitable to be classified by soft clustering analysis. In hard clustering analysis such as k-means, each gene can only be divided into one cluster. Soft clustering algorithm, also known as fuzzy clustering, is different from hard clustering in that it can divide the samples in the datasets into one or more clusters with a certain probability and is more applicable for datasets that are hard to be distinguished and can decrease noise [18].

## 3. Results

### 3.1. Differential Expression Analysis and Functional Enrichment Analysis

**Figure 2:**
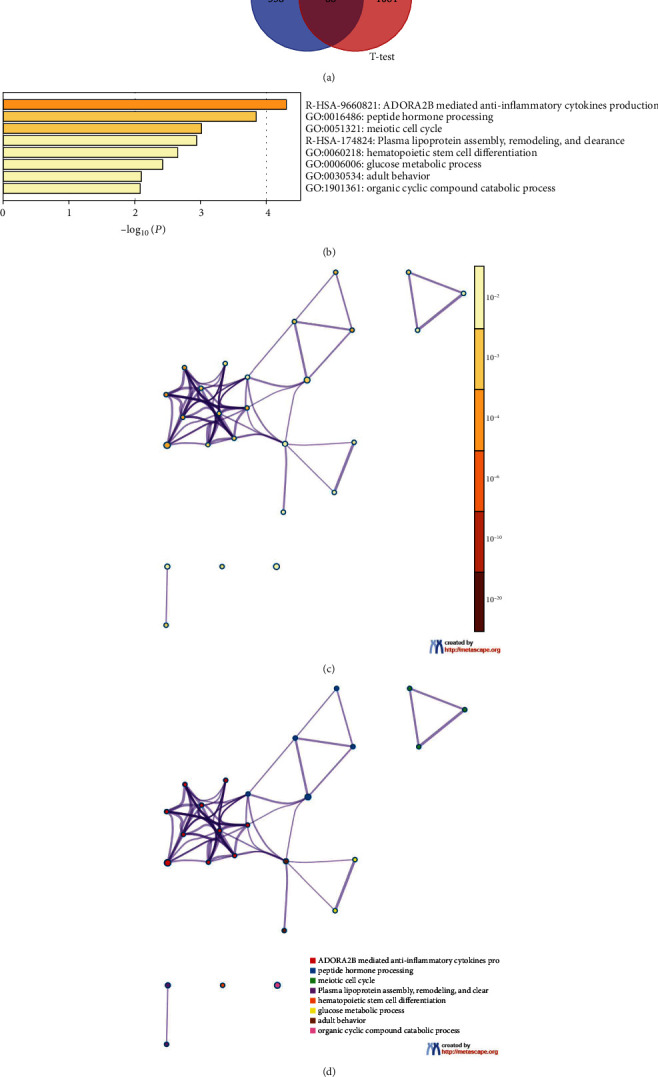
Differential expression analysis and functional enrichment analysis. (a). Venn diagram of DEGs of PS, MSO, and VSO patients (ANOVA test) and DEGs of VSO and VSOC (*t* test). (b) Bar chart of Metascape enrichment analysis (enriched terms are ranked by *p* value; deeper color of the bar chart denotes smaller *p* value; smaller *p* value denotes higher ranking). (c) Metascape enrichment network displayed by *p* value. Smaller *p* value presents deeper nodes color. (d) Metascape enrichment network displayed by terms (different colors denote different terms; nodes with the same color belong to the same term; large nodes include more genes; and the thicker the line between nodes, the higher the correlation).

The expression profiles of patients with varying OSA severities were analyzed by ANOVA test (*p* < 0.05), and a total of 446 DEGs were screened. (Supplementary Table 1). DEGs of VSO and VSOC patients were analyzed using *t* test. A total of 1,152 DEGs were obtained (*p* < 0.05) (Supplementary Table 2). By overlapping the two groups of DEGs, a total of 88 DEGs reflecting OSA severity were finally determined (Figure 2(a)) (Supplementary Table 3). To analyze the biological functions and signaling pathways these DEGs involved, Metascape enrichment analysis was conducted. It was shown that these genes were mainly enriched in ADORA2B mediated anti-inflammatory cytokines production, plasma lipoprotein assembly, remodeling, clearance, and glucose metabolic process (Figures 2(b)–2(d)).

### 3.2. Soft Clustering for the DEGs Related to OSA Severity

**Figure 3:**
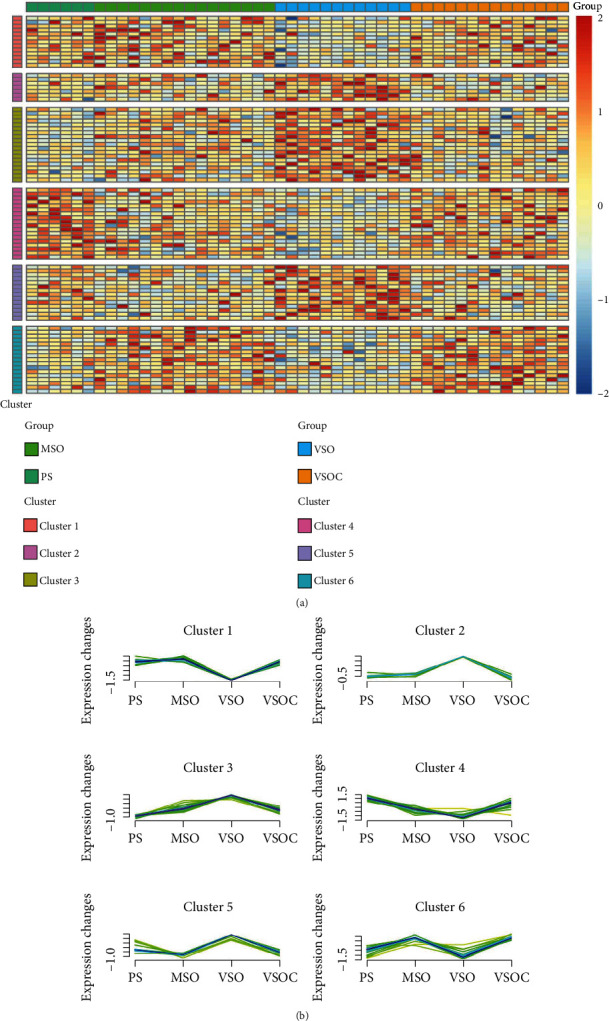
Soft clustering analysis. (a). Heatmap of gene expression in soft clustering analysis (*x*-axis: different severity OSA patients; *y*-axis: 6 clusters). (b) The expression changes of genes in 6 clusters of patients in different stages (green and yellow curves: gene expression mode with low acore; red and purple: gene expression mode with high acore; higher acore presents closer gene expression mode is to that of CLUSTER).

Mfuzz package was utilized to cluster the samples based on the 88 DEGs, by which obtained 6 clusters (Supplementary Table 4). The expression of genes in cluster 3 elevated as severity increased while dropped by treatment. The expression of genes in cluster 4 dropped as disease progressed, but the expression was elevated after treatment (Figures 3(a)–3(b)). The expression trend of the other clusters showed no relevant trend with disease severity. Therefore, we focused on genes in cluster 3 and cluster 4 in the next step.

### 3.3. Identification of Cluster Marker Genes

**Figure 4:**
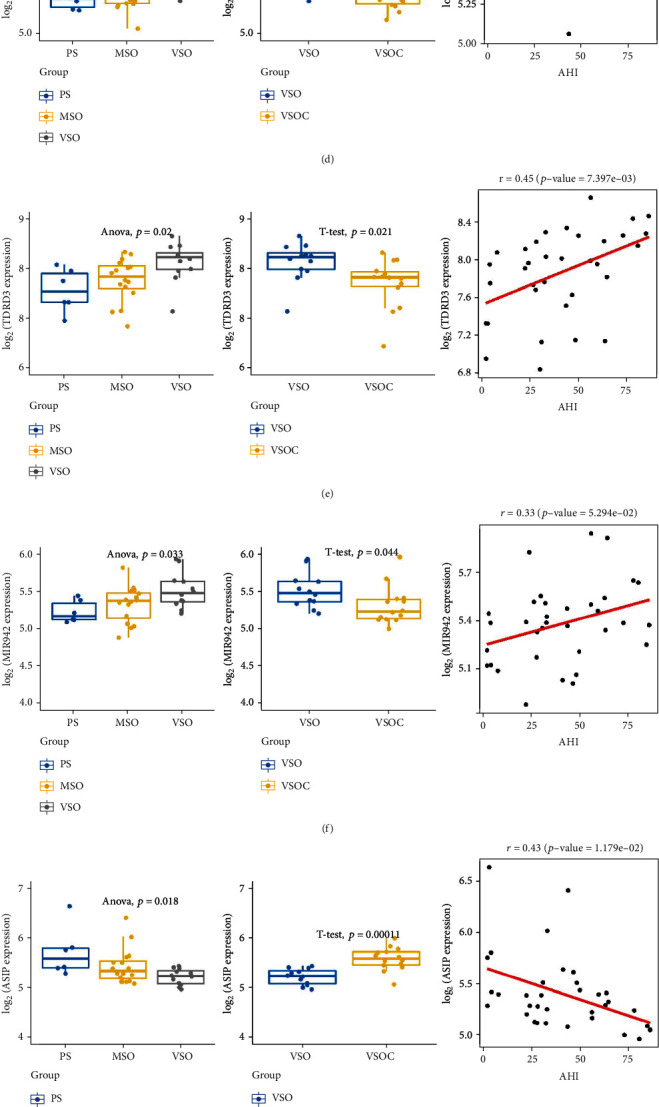
Expression of cluster core genes and correlation analysis. (a–j) The expression of all cluster marker genes in PS, MSO, VSO, and VSOC groups was analyzed. Pearson correlation analysis was undertaken on these genes and AHI.

Cluster marker genes from both of the above clusters were selected (acore > 0.6), by which *KCNN2*, *MIR942*, *PLXNB3*, *SERPINA12*, *TDRD3,* and *UPK1B* were screened from cluster 3, also *AASS*, *ASIP*, *LINC00494*, and *SAXO2* were screened from cluster 4. Subsequently, the expression of screened genes in different severity of patients (PS, MSO, VSO, and VSOC) was determined based on GEO-derived dataset, followed by Pearson correlation analysis with AHI. AHI is considered the most objective index for measurement of OSA severity. The expression of *KCNN2*, *MIR942*, *PLXNB3*, *SERPINA12*, *TDRD3*, and *UPK1B* significantly elevated as OSA progressed, while significantly dropped after treatment. Except for *MIR942*, the expression of above 5 genes was significantly positively correlated with AHI (Figures 4(a)–4(f)). The expression of *AASS*, *ASIP*, *LINC00494*, and *SAXO2* significantly declined as OSA progressed while significantly elevated after treatment. These gene expression levels were markedly negatively correlated with AHI (Figures 4(g)–4(j)). Taken together, cluster cores in cluster 3 could indicate an increasing OSA severity, while those in cluster 4 were potentially related to the reduced severity of OSA.

## 4. Discussion

Some disease-related gene studies conducted a series of microarray analyses based on DEGs in different severity of specific diseases. For instance, in a study on bipolar disorder (BD), Ya-Chin Lee et al. [19] undertook differential expression analysis on RNA expression profile from blood samples of manic and remission patients. Lastly, encoding genes *TAS2R5* and *TASER3* and some lncRNAs and miRNAs (MIR181B1 and MIR103A1) were found to be gene markers of BD manic episode via differential expression analysis [19]. Similarly, this study further analyzed DEGs related to different severity of OSA and finally determined gene markers to reflect OSA severity. Compared to the study mentioned above, this study adopted similar approaches to dig DEGs reflecting OSA progression despite different classification methods.

It is well known that OSA elevates risk of cardiovascular disease in adults [20]. A theory pointed out that OSA-derived systemic inflammatory response triggers cardiovascular disease [21, 22]. In this theory, OSA first causes intermittent hypoxia hypercapnia (IH) in patients, and then IH activates patient's systemic inflammatory response via HIF-1 or NF*κ*B [23, 24]. All the above, inflammatory response-related biological functions or signaling pathways play a vital role in OSA course. In this study, Metascape enrichment analysis displayed that different severity of OSA-related DEGs was mainly enriched in ADORA2B mediated anti-inflammatory cytokines production. Therefore, these DEGs were believed to participate in biological functions related to inflammatory response.

In gene expression profile analysis, researchers are often concerned about expression profile characteristics changing with time sequences. For example, the expression mode of iron death-related genes was analyzed during hepatocellular carcinoma progression by Zuo Fei et al. [25]. A Chinese research team investigated survival-related genes in primary glioma via microarray analysis [26]. These studies all used Mfuzz package to perform soft clustering analysis on expression profile data. Soft clustering analysis is not only suitable for gene dynamic mode changing with time. This method can also be applied to analyze gene expression characteristics of diseases of varying severity. For example, Hongyun Qin et al. [27] used Mfuzz package to undertake soft clustering analysis on DEGs of mild cognitive impairment and Alzheimer's disease to explore corresponding biomarkers. However, there is a lack of studies on OSA-related expression genes at different severity. Although Xiandong Gu et al. [28] conducted functional enrichment analysis and protein-protein interaction (PPI) analysis based on DEGs of gene expression profile of visceral adipose tissue OSA patients and normal people, they also determined main functional subset in PPI network during OSA pathogenetic process. However, they only focused on genes related to OSA onset. Differently, our study conducted soft clustering analysis on DEGs based on the gene expression profile of OSA at different severity, and finally screened out the marker genes reflecting OSA disease changes.

A recent study concluded that OSA-induced chronic intermittent hypoxemia leads to cognitive impairment through inflammatory responses [21]. Cluster marker gene *KCNN2*, which encodes potassium calcium to activate pathway protein, was screened out in this study and played an important role in neural network function [29]. A study published in Nature Neuroscience in 2020 showed that *KCNN2* expression in mouse nerve cells is associated with motor learning disabilities [30]. In addition, Fumiya Tatsuki et al. [31] reported in a study published in Neuron that impaired *KCNN2* leads to shorter sleep duration. On the whole, it is posited that *KCNN2* is crucial in the regulation of neural networks in sleep and may also be altered during OSA-induced cognitive impairment and other neurological injuries.

Taken together, this study accessed expression profile data in different severity of OSA from GEO public database and screened relevant gene markers via differential expression analysis and soft clustering analysis. Next, we detected the expression of these genes in OSA patients at varying severity and those before and after treatment. Correlation between these gene expressions and AHI was tested. Altogether, *KCNN2, PLXNB3*, *SERPINA12*, *TDRD3*, *UPK1B*, *AASS*, *ASIP*, *LINC00494*, and *SAXO2* (significantly correlated with AHI) are expected to be gene markers for OSA severity. Despite comprehensive microarray analysis used in this paper, limitations still existed. There was a lack of mechanism studies; thus, we could not explain the specific role of these genes in the progression of OSA. Therefore, we plan to construct OSA mouse model and conduct molecular experiments and cellular experiments for further analysis of major action mechanism of these genes.

## Data Availability

The data used to support the findings of this study are available from the corresponding author upon request.

## Supplementary Materials

Supplementary 1 Supplementary Table 1. DEGs of OSA samples with of varying severity.

Supplementary 2 Supplementary Table 2. DEGs of OSA samples before and after treatment.

Supplementary 3 Supplementary Table 3. Genes in the overlap of two groups of DEGs.

Supplementary 4 Supplementary Table 4. Six clusters obtained by soft clustering analysis.
