# Supplementary material for: Identification of the Novel Gene Markers Based on the Gene Profile among Different Severity of Obstructive Sleep Apnea
Source: Comput Math Methods Med. 2022 Oct 4;2022:6517965. doi: 10.1155/2022/6517965 (PMC9554663; doi:10.1155/2022/6517965)
Supplement: Supplementary 1 — Supplementary Table 1. DEGs of OSA samples with of varying severity. [file 6517965.f1.pdf]

ID	ANOVA
KRTAP5-9	0.000224
LOC10013	0.000278
LINC0063	0.000321
C4orf45	0.000424
SCN1A	0.000497
PHGR1	0.00061
LOC28439	0.000612
CCDC163	0.001016
NUDT4	0.001083
PARD3B	0.001396
GNG13	0.00144
C3orf20	0.001637
SLC17A7	0.001722
SH3BGR	0.001739
MIR1295A	0.002185
FNDC3B	0.002222
LIN28B	0.002243
TRPA1	0.002344
OR2C3	0.002377
WFDC10A	0.002489
IQCA1	0.002527
TTC36	0.002579
NF1P2	0.002789
SNORD11	0.003096
EXD1	0.003102
NRAD1	0.003246
KRBOX4	0.003327
OR51B5	0.003328
PROB1	0.00336
PCSK1N	0.003375
C1orf189	0.003877
SNORD11	0.003972
ATRNL1	0.004024
FLRT1	0.004178
CACNB4	0.004253
SYCP1	0.004346
TAGLN3	0.004534
SLC13A3	0.004727
TAS1R1	0.004815
WFDC5	0.005145
CFAP57	0.005344
MS4A10	0.005416
GHRHR	0.005424
PLGLA	0.005551
FAM149A	0.005553
AP1S3	0.005561
SERPINA1	0.006215
ADAMTS2	0.00627
TFAP2D	0.006325
ADRB1	0.006475
GPR32	0.006544
ZNF295-A	0.006597
TAAR1	0.006777
MIR422A	0.006806
BRD3OS	0.006891
CHST11	0.007202
USF3	0.007252

STOML2	0.007362
LINC0030:	0.007416
ADAMTS2	0.007461
LOC44089	0.007501
GPRASP1	0.007538
BRCA2	0.007735
SCT	0.007747
HJV	0.008173
LOC34035	0.00831
WDR72	0.008519
AASS	0.00854
TMEM191	0.00885
GALM	0.008894
TRIM60	0.008954
TOPAZ1	0.009205
MXRA5	0.009247
OR5A2	0.009306
GJD2	0.009515
LDB1	0.009535
FOXL1	0.00969
PROSER3	0.00984
OR8G1	0.010018
LRRC74B	0.01011
CALCB	0.010365
OR1E2	0.010493
SERPINA3	0.010954
SNORD11	0.010976
MS4A6E	0.010984
CYP2A7	0.011219
MPG	0.01125
UCN3	0.011388
CRYBA4	0.011426
MLANA	0.011571
DIRC1	0.011624
SULT2A1	0.01176
SAMD7	0.011842
PLXNB3	0.011893
LRIT3	0.011962
IL12RB2	0.012058
HOXB1	0.012071
GNRHR2	0.012179
OR4S1	0.0122
NR2F1-AS	0.012342
SVEP1	0.012536
CASP14	0.012544
ARL17A	0.012565
PGS1	0.012639
TMCC3	0.012718
SAXO2	0.012761
LCE3A	0.012813
HAND2-A	0.013017
ADCY4	0.013094
MIR499A	0.013237
PRAMEF2	0.013294
CRNN	0.01356
TBC1D22E	0.013797
RPS6KL1	0.01391
GUSBP14	0.01406

FABP2	0.014279
NAP1L2	0.014301
L3MBTL4	0.014377
RUSC1-AS	0.014467
KCTD10	0.014577
SH2D3A	0.014595
RNASE8	0.014601
NECAB1	0.014657
PIN1P1	0.014785
CCL4	0.014833
SLC12A6	0.014926
SNTA1	0.01515
CPEB1	0.015379
CIT	0.015417
ADPGK	0.015525
SLC22A8	0.015634
MIR298	0.015748
HOXB-AS:	0.015762
AP5B1	0.015793
RPS27	0.016065
MIR553	0.016182
PAPOLB	0.016257
LMTK3	0.016327
GM2A	0.016351
APLNR	0.016375
GRM3	0.016475
OPRD1	0.016585
A1BG-AS1	0.016758
SNORD45	0.017041
PROM2	0.017121
GPR182	0.017246
BHMT2	0.017366
TULP2	0.017828
SYT1	0.0179
ASB16-AS	0.018019
ASIP	0.018122
NEUROG3	0.018318
CEL	0.018563
CUBN	0.018601
TMEM221	0.018666
SNHG28	0.018776
NKX1-2	0.018828
CYP7A1	0.018943
HSFY1P1	0.01921
FOXH1	0.019243
SCN3B	0.019433
PRAP1	0.019528
PSMB11	0.019549
GLRA3	0.019636
TUBGCP2	0.019647
DUSP26	0.019836
TRIM49D2	0.020017
CCDC9	0.020118
NCAM2	0.020154
TDRD3	0.020187
ARHGEF37	0.020676
NPSR1	0.020753
UBXN11	0.020791

KCTD7	0.020876
LRRIQ3	0.021029
FBXO40	0.021158
MIR146B	0.021174
TTC9B	0.021237
PPP2R2C	0.021445
TONSL	0.021573
KCTD16	0.021627
SLFNL1-A	0.021743
ACE	0.021884
ZNF516	0.022378
CATSPERZ	0.022456
SUSD5	0.022497
CIDEC	0.022634
INSR	0.022755
FAM184B	0.022839
SSX2IP	0.022857
OR52E6	0.022933
PIGK	0.023123
HAVCR1	0.023289
TMEM52	0.02354
CCDC85A	0.023596
FBXL16	0.02387
OR10A4	0.023904
MIR921	0.024017
DNASE2B	0.02407
PPP1R36	0.024239
C7	0.024253
CD209	0.024305
RPS6KA6	0.024492
ZC3H7B	0.024511
PRXL2A	0.024562
CCDC38	0.024571
KIF9	0.02464
CYP2W1	0.02475
AKAIN1	0.024811
LIAS	0.025048
EEF1AKMT	0.025049
KRT6A	0.025078
BPESC1	0.025262
RSPO4	0.025429
RUNDC3A	0.025457
MIR548O	0.025474
CPE	0.025676
COL17A1	0.025939
DSTYK	0.026441
MZT2A	0.026488
TCERG1L	0.026621
EXOSC8	0.026688
IL1RAPL2	0.026708
NDUFB2-/	0.026803
STARD7-/	0.026849
UBR2	0.0269
OR4A16	0.027392
LOC39219	0.027514
AMOTL2	0.027797
DAZ2	0.027919
LINC0220	0.027983

CRCT1	0.02805
IQCF5	0.028301
UPK1B	0.028314
UAP1	0.02834
SLC22A11	0.028391
CLCN1	0.028481
C3orf67	0.028608
PLEKHA7	0.028622
ESS2	0.028774
GPR37L1	0.028825
KCNQ2	0.028896
PRDM6	0.028962
THPO	0.029143
MRPL47	0.029344
ZNF704	0.029544
CXCL14	0.029655
USP49	0.029691
PLEKHG4E	0.02991
KCNN2	0.030247
LINC0049	0.030248
PTOV1-AS	0.030328
XKR4	0.030388
NAV3	0.030432
LOC40071	0.030449
CRIP2	0.030616
FGF1	0.030661
LOC10013	0.030985
SRD5A3	0.031123
LSP1P3	0.031155
DIPK2B	0.031163
NKX2-1	0.031264
FGD5P1	0.031291
LINC0048	0.031508
HSP90AA6	0.03176
ASB10	0.03178
SRD5A2	0.032019
RPA1	0.032347
MAS1	0.032391
PALM2AK	0.03259
MIR942	0.032713
MUC2	0.032854
BBS1	0.032981
ISM2	0.033198
TIAM2	0.033229
SPDYE7P	0.033247
GLP1R	0.033319
GCK	0.033535
PEX5L	0.033654
MIR302E	0.033701
LENEP	0.033834
WFDC3	0.034076
ZBTB11	0.034594
FZD9	0.034653
ZFP41	0.034761
EPHX2	0.034772
CPD	0.034858
LINC0061	0.034881
TUSC7	0.035224

OR4N3P	0.035288
RPL22	0.035697
PRUNE2	0.035728
FNDC1	0.035821
LINC0112(	0.035867
ZNF790	0.035887
SCGB1D4	0.035915
NPY1R	0.035924
ORC6	0.035959
GOLGA2P	0.035965
CPNE4	0.035967
LOC10013	0.036189
LOC10028	0.036233
PRMT1	0.036297
ALS2	0.036309
SNORA36	0.036461
C1orf68	0.036492
NEIL2	0.036537
PLTP	0.036839
DCC	0.036847
SMC1A	0.036885
IL31	0.03692
ZNF705A	0.036944
TMEM215	0.036971
MIR548A3	0.03698
AIRE	0.036987
FKBP7	0.037011
SIX3	0.037057
NFE4	0.037149
LOC10012	0.037207
MAB21L4	0.037231
CADM4	0.037259
SNORD79	0.037314
ZNF705B	0.037352
SUN3	0.037472
DKK4	0.037528
NR0B2	0.037625
TENM1	0.037636
MIR612	0.037902
DNASE1L3	0.037938
ADGRB2	0.038011
LINC0154(	0.038275
C22orf42	0.038362
PFKFB1	0.038428
ETFBKMT	0.038438
LINC0140!	0.03846
HEPACAM	0.038794
LOH12CR2	0.038802
C6orf58	0.038849
PRAMEF1(	0.039029
VARS2	0.039104
HMGN3	0.039168
FZR1	0.039266
TFDP2	0.039406
TLDC2	0.039752
MIR1258	0.039838
ZNF12	0.039918
LTK	0.040049

CEACAM1	0.040055
DEAF1	0.040228
BPHL	0.040289
PREB	0.040392
EBF4	0.040442
ZBTB37	0.040593
MIS18A	0.040696
TTC39C	0.040922
ABHD14B	0.041
SH3GL3	0.041062
CACNG4	0.041264
CNP	0.041381
TMEM190	0.041522
EEF1G	0.04163
OR4K1	0.04167
MIR595	0.041702
FAM71F2	0.041717
SH3GL1P2	0.04172
OR10G8	0.041734
MIR637	0.041884
OR8H1	0.042239
MTF2	0.04225
EBLN1	0.042363
RASA4CP	0.042884
MEP1B	0.043014
ASIC2	0.043025
USP29	0.04312
FAM221A	0.043169
METTL8	0.043202
FAM197Y2	0.043267
FOXA2	0.0433
RIPPLY2	0.043461
CCBE1	0.043467
MIR187	0.043471
MIR34A	0.043495
FBXL19	0.043608
MIR210	0.04369
MIR1227	0.043831
APEH	0.043854
RPL18A	0.043953
OSR2	0.044102
NUAK1	0.044117
OR8S1	0.044211
XIST	0.04431
NOP56	0.04432
AQP3	0.044323
ALX4	0.044385
OR2V2	0.04448
CRB3	0.044511
WNK3	0.044527
SCGB2B2	0.044546
TRABD2A	0.044611
FAM161B	0.044614
LINC0203[illegible]	0.044616
IGLON5	0.044635
PCDHB8	0.044881
UGT1A8	0.045026
SHH	0.045359

NEUROG2	0.045692
PSD3	0.045928
NEK3	0.045931
ANXA10	0.045932
RAF1	0.046045
RBMY3AP	0.046132
ZC3H7A	0.04633
LUM	0.046417
TMEM106	0.046646
GUF1	0.046768
SPTLC1	0.046775
STK33	0.046879
1-Dec	0.046918
ZNF766	0.047148
KRTAP12-	0.047308
TUBA3C	0.047313
LILRB3	0.047421
MLLT3	0.047525
HDDC3	0.047526
B3GNT3	0.047687
MAPK8IP2	0.047748
RNASE11	0.047876
KLK8	0.047893
SLFN12L	0.047924
OR4E2	0.048056
SMG1P7	0.048061
WNT11	0.048073
SAR1A	0.048085
TUBBP5	0.048139
C10orf90	0.048398
TXLNB	0.048553
ECRG4	0.048684
IL15RA	0.048741
LINC0066[illegible]	0.048777
MT1JP	0.048861
LINC0120[illegible]	0.049111
OR10Q1	0.049209
SYAP1	0.049258
AIPL1	0.049368
FAM106A	0.049419
UCK1	0.049779
