# Supplementary material for: Identification of the Novel Gene Markers Based on the Gene Profile among Different Severity of Obstructive Sleep Apnea
Source: Comput Math Methods Med. 2022 Oct 4;2022:6517965. doi: 10.1155/2022/6517965 (PMC9554663; doi:10.1155/2022/6517965)
Supplement: Supplementary 2 — Supplementary Table 2. DEGs of OSA samples before and after treatment. [file 6517965.f2.pdf]

ID	T-test
PTPN20	4.77E-05
ASIP	0.000107
ZNF146	0.000157
NLRP14	0.000167
NSFL1C	0.000182
ZSCAN25	0.000208
RTEL1-TN	0.000286
SLC2A12	0.0003
FAM215A	0.000334
SNORD11	0.00039
CNP	0.000392
TMA7	0.00056
PPBPP2	0.000577
OSGEPL1	0.000715
RRM2B	0.000784
FAU	0.000876
SDAD1	0.000878
GOLGA5	0.00095
ONECUT1	0.00129
SGMS1	0.001321
NRL	0.001325
EML2	0.001357
ADCY4	0.00157
CRYBA4	0.001594
ZNF704	0.001608
GPR61	0.00163
RNF112	0.001763
SERTM1	0.001773
NBEA	0.001836
TCF12	0.001838
COL4A4	0.001842
RPA1	0.001961
PCDHA5	0.002224
C3orf35	0.002245
SSB	0.002435
OR6C65	0.002659
MSGN1	0.002714
TFCP2	0.002796
NPSR1	0.002882
MORF4L1	0.00293
ZNF71	0.002945
MAP3K14	0.002979
SIGLEC17P	0.002985
NRADDP	0.003084
ZNF681	0.003131
LINC0227	0.003132
MRC1	0.003166
WDR92	0.003374
GATAD2A	0.00339
RSPO4	0.003441
CIDEC	0.003468
LOC72802	0.003476
BAP1	0.00351
ZNF232	0.003567
ATIC	0.003573
PTPN9	0.00359
LOC10012	0.003601

TMEM252	0.003637
POU3F1	0.003699
VPS9D1	0.003752
CALCR	0.003773
MIR645	0.003802
SUSD5	0.003883
KRIT1	0.004031
USP27X-A	0.004035
RAB40A	0.004184
RNF20	0.004187
LINC0049	0.004235
MRPL33	0.004246
SVOP	0.004312
MIR2052H	0.004429
HLA-J	0.004585
BMP7	0.004604
API5	0.004609
CDKL5	0.004612
DUSP26	0.004693
CACUL1	0.00472
LOC10012	0.004747
SNORD11	0.004766
DUSP13	0.004771
CLCF1	0.004799
STX18	0.004812
TRERF1	0.004986
ROCK1P1	0.004989
LINC0063	0.005002
MIR323A	0.005042
TERF1	0.005134
LRRC40	0.005411
ZNF396	0.005424
HNRNPF	0.005427
RPL35A	0.005498
CTRL	0.005498
KCMF1	0.005611
ARCN1	0.005646
FAM21EP	0.005846
COX4I2	0.005884
MIR1245A	0.005993
SMAD7	0.006009
OR51B5	0.006044
CEP41	0.006058
FSD1	0.006065
FGF18	0.006133
SPPL3	0.006159
RAB6C-AS	0.006256
PHGR1	0.006391
SPEM2	0.00641
NUDT14	0.006428
ATP5F1E	0.006505
MRPL50	0.006519
FAM197Y2	0.006644
PHF2	0.006648
ARMCX3	0.006698
RNF180	0.006705
MTHFR	0.006708
UFSP2	0.006754

RHO	0.006768
SMPD3	0.006809
CASC16	0.00683
SNORD11	0.00685
DICER1-A	0.006886
FOXD2	0.006889
CTSLP2	0.007069
FEM1C	0.007094
IPO7	0.007102
STT3B	0.007121
PREPL	0.007151
STAC2	0.007168
GSTM4	0.007183
ELAVL3	0.0072
OR1S1	0.007238
HUS1B	0.007242
REEP3	0.007255
SLC35F3	0.007276
CDH3	0.007354
ANP32B	0.007359
ACVR2B	0.007408
MRPS33	0.007466
DLX1	0.007474
SURF2	0.007566
PATE4	0.007605
EXTL3	0.007633
NXF3	0.007655
MARCHF1	0.007664
MIR300	0.007685
SHQ1	0.007787
ZBTB39	0.007894
FAM98A	0.007986
SVEP1	0.007991
SRPRB	0.008004
GTF2I	0.008074
MYLK3	0.008096
GUCA1B	0.008144
MCCC2	0.008162
CEMIP	0.008194
SNORA1	0.008214
RAD9B	0.008309
MRPS14	0.008348
CPNE4	0.008401
MIR539	0.008456
PICK1	0.008601
TMC3	0.008848
C15orf61	0.008864
MIS18A	0.008974
GPD2	0.009015
TMEM255	0.009045
NCAM2	0.00907
ZNF527	0.009118
ATF6	0.009161
NUTM2A	0.009219
LOC10028	0.009231
PLCB4	0.009336
MYSM1	0.009364
FIS1	0.009369

CD209	0.009398
KLK13	0.009448
TFAM	0.00947
TGM5	0.009554
RPGRIP1L	0.009572
CUBN	0.009598
P3H2	0.009756
ACADM	0.009769
RPL32	0.009858
PDE1B	0.009973
TMEM215	0.010002
FOXH1	0.010011
EIF3A	0.010019
SNORA74	0.010037
ACTL6B	0.010057
SEC22B	0.010061
MYOG	0.010118
LIMA1	0.010146
MTREX	0.01017
PDE4A	0.010195
DAPK2	0.01024
PTCD3	0.010263
PIP	0.010334
GATC	0.010365
STK24	0.010403
B3GNT7	0.010479
MAN2A2	0.01057
HOXA-AS	0.01063
PCDHGB8	0.010657
ELL	0.010775
HES6	0.010821
LOC64269	0.010891
GPR171	0.010912
SETD5	0.011005
AMER3	0.011076
TRIM65	0.011144
GET3	0.011146
FAM193B	0.011242
VDAC3	0.011352
FARSB	0.011356
CASTOR2	0.011366
AMIGO2	0.011382
CDK9	0.011394
LPA	0.011434
FLJ46906	0.011474
TBKBP1	0.011492
UBQLN2	0.011494
KPNA3	0.011515
C14orf119	0.011519
EIF2B2	0.011654
JAZF1-AS1	0.01167
C7orf25	0.011751
MYOM2	0.01185
LBR	0.011915
IMMT	0.011954
LOC28439	0.011973
HEXIM1	0.012053
THAP2	0.012182

CCDC144A	0.012278
NPY1R	0.012295
SFSWAP	0.012352
MYO9B	0.012382
PKN1	0.012417
STARD7-A	0.012453
NBEAL2	0.012547
RPS3	0.012547
CAND1	0.012646
TUSC7	0.012718
CEL	0.012782
AJAP1	0.013108
TLDC2	0.013148
FRMD4B	0.01317
GRID1-AS	0.013241
ICAM1	0.013406
PRR25	0.013501
COPS8	0.013525
SPTBN5	0.01353
MIB1	0.013565
SAXO2	0.013606
FOPNL	0.013657
CEP295NL	0.013663
MIR486-1	0.013714
PRODH2	0.013938
GPRASP1	0.014015
OR6K3	0.014041
VAMP7	0.014049
LLGL1	0.014066
NET1	0.01407
RDH11	0.014096
MIR758	0.014155
C8orf34	0.014241
CNEP1R1	0.014358
LINC0093	0.014388
TIAM2	0.014496
ALDH6A1	0.014511
KCNJ10	0.014515
SLC66A1L	0.014529
AKT2	0.01453
MIR208B	0.014532
ERGIC2	0.014564
SRD5A3	0.014625
LINC0114	0.014698
CAPSL	0.01476
GHRHR	0.014761
IGHMBP2	0.014767
HIVEP3	0.014812
OASL	0.014823
LINC0026	0.014845
RUNX1T1	0.014953
CCDC144	0.015033
NMUR2	0.01513
LOC10012	0.015158
MZF1-AS	0.015215
SLC16A7	0.015405
CASZ1	0.015446
BET1	0.015475

DAAM1	0.015493
OR14C36	0.015566
SCARNA1(	0.015611
NOL12	0.015637
IFFO2	0.015652
ZNF189	0.015664
RGPD8	0.015724
KDM2A	0.015733
TSPY26P	0.015746
AKAP17A	0.015762
CLDN6	0.015853
AASS	0.015855
SYCP1	0.015911
OR10J3	0.015926
STARD10	0.016029
ZNF550	0.016036
OR4N3P	0.016254
F8	0.016267
TMCC3	0.016381
INTS13	0.016429
TMOD2	0.016438
PHLPP2	0.016489
HS6ST1	0.016518
PFKFB3	0.01658
ACSM5	0.016617
PAAF1	0.016724
FBXO5	0.016742
KLK7	0.016822
MIR361	0.016895
COPB1	0.016963
TMEM168	0.016981
KCNH3	0.016989
GZF1	0.017024
NOTUM	0.017101
RGMB-AS	0.017146
FBXO33	0.017158
PRIM1	0.01717
POT1	0.017204
CTCF	0.017209
ARMC8	0.017293
RRM1	0.017315
LOC10012	0.017357
VBP1	0.017375
FAM53B	0.01742
HCN4	0.017451
SEMA4C	0.017465
AQP4	0.017471
CACNA2D	0.017531
NARF	0.017544
EHHADH	0.017565
PRAMEF14	0.017573
NXNL1	0.017598
TENT5D	0.017638
PRSS30P	0.017648
CCAR1	0.017732
RMND5A	0.017752
NSD1	0.017787
DNAJC24	0.017898

POM121L1	0.017939
LINC0089!	0.017965
TRIM61	0.018083
NCBP2	0.01811
ARMCX5	0.018194
LGR6	0.018195
VSTM1	0.018223
NAP1L1	0.018264
REM2	0.018309
MICALL2	0.01831
METAP1	0.018329
PLPP6	0.018342
TMEM207	0.018367
POM121	0.018441
ADGRG4	0.018501
PPP2R2D	0.018561
STXBP6	0.018711
PLEKHG3	0.018738
METAP2	0.018743
PRDX6	0.018766
TMEM255	0.018847
FRS2	0.018863
IQCN	0.018874
TUBA3C	0.018916
PCYT2	0.019063
YIPF4	0.01912
EIF3CL	0.019149
PRDM14	0.019269
NOTCH2	0.019374
CNBD1	0.019377
MINDY2	0.019427
PRIMPOL	0.019429
OR7G2	0.019495
YME1L1	0.019588
BRPF3	0.019805
LDB1	0.019824
SLC23A2	0.019846
NBDY	0.019905
PAGE2	0.019917
ZNF543	0.019987
DMXL1	0.019992
SPINK6	0.02003
TYW5	0.020033
SAFB2	0.020063
SPRR4	0.020072
CMTM1	0.020081
CNOT6	0.020218
STAG3L4	0.020251
CXorf58	0.020266
NDNF	0.020322
UPK1A	0.020323
U2AF2	0.020362
GRM8	0.020412
CCDC96	0.020439
CHST2	0.020455
DDAH2	0.020466
POPDC2	0.020547
CPEB4	0.020596

GPR153	0.020634
RAB11B	0.020638
SCO2	0.020694
MED26	0.020715
UPK2	0.020741
GTF2F2	0.020783
SLC13A1	0.020823
METTL18	0.020871
CHRNA1	0.020872
DYNLRB1	0.020898
SPACA5	0.020928
OTX2	0.020962
RBM34	0.020986
C3orf49	0.021012
HGH1	0.021137
TBC1D5	0.021143
PIGK	0.021208
TDRD3	0.021234
ACE	0.021253
CASP2	0.02127
LIN52	0.021282
NLRP4	0.021356
IDUA	0.021358
TMEM120	0.021372
KAZN	0.021395
RNF41	0.021669
NT5C3A	0.021715
FAM104B	0.021783
PXT1	0.021784
LINC0112	0.021819
KDM4B	0.021866
BBS12	0.021895
ARSH	0.02195
ABCG5	0.022007
KIF16B	0.022009
RPL10L	0.022024
LCE3A	0.022048
LDHB	0.022063
MRPL51	0.022272
PDCD11	0.022281
SLC22A9	0.022293
TPRXL	0.022325
FOLR2	0.022372
NOTCH1	0.022374
ATP5F1EP	0.022404
ZCCHC3	0.022434
NPM1	0.022438
TBL1XR1	0.022517
KDM6A	0.022547
CNTF	0.022571
GATA5	0.022575
TMOD3	0.022626
GET1	0.022649
ECI2	0.022695
CAPRIN1	0.02276
HNRNPKP	0.022802
SFR1	0.022821
PSMA5	0.022836

ERBIN	0.022837
CLASRP	0.02287
TVP23A	0.022931
TMEM82	0.022947
FAAP20	0.02295
PCNA	0.023097
HSPH1	0.023131
NR2E3	0.023235
FNDC4	0.023254
MRS2	0.023304
DENND5A	0.023335
BCAM	0.023379
MIR598	0.023435
EXOSC8	0.023443
TP73-AS1	0.023469
NUDT7	0.023473
G3BP1	0.023511
SLC6A14	0.023554
CCT8	0.023565
TBC1D14	0.023568
COPS4	0.023579
LRWD1	0.023614
RBL1	0.023659
PHKG2	0.02369
UBOX5	0.023692
TCTN3	0.023724
TIMM9	0.023811
TRAK1	0.023918
MRGPRD	0.024
EFHD2	0.024003
PROB1	0.024188
RPS15	0.024328
MXRA8	0.02435
FAM83C	0.02443
BFAR	0.024456
SNORD42	0.024547
SRXN1	0.024554
PARP1	0.024556
U2SURP	0.024597
CTAGE6	0.024603
AVIL	0.024605
TMEM221	0.024619
ZNF37A	0.024622
STK11IP	0.024683
TMEM155	0.024699
TMEM86A	0.024702
GLOD4	0.024728
RIPK2	0.024904
FAM219A	0.024918
METTL2A	0.025251
MIR639	0.025283
OR4A16	0.025313
PITHD1	0.025465
FAM83F	0.025569
ELP4	0.02559
STK25	0.025595
CXorf56	0.025639
RPL22L1	0.025692

DCLRE1A	0.025714
NOP56	0.025814
FOXA2	0.025856
PTGES3	0.025879
STAG3	0.025943
ARHGEF1(	0.025973
LRRN4	0.026031
FASTKD5	0.026087
LINC0112:	0.02611
MVP	0.026131
PSMD14	0.026151
SLC22A25	0.026163
RRAD	0.02618
GRIK5	0.026188
DDIT4L	0.026214
IL3	0.026216
SNORD11	0.026231
ZKSCAN3	0.02628
STAG2	0.026374
RPE	0.026412
PNKP	0.026418
DCUN1D1	0.02648
THBS3	0.026494
CRADD	0.026588
ADM2	0.026598
C20orf173	0.026687
PLP1	0.026706
DNASE1L3	0.026741
SF3B3	0.026744
TMEM196	0.026782
HOXD1	0.026807
DYNLL1	0.026878
ACTR10	0.026886
HSPD1	0.026917
ZNF679	0.027006
SLC45A4	0.027023
TRNP1	0.027044
ANKRD18/	0.027135
PLXNA4	0.02721
SUMF1	0.027236
SPATA42	0.027246
SLC25A28	0.027258
PI4KAP1	0.027288
ZNF276	0.027343
DEPDC1B	0.027429
THBS2	0.027459
CPSF6	0.02746
SETD2	0.02747
SNORD50	0.027529
HELZ2	0.02755
LCORL	0.027564
SLC39A6	0.027583
SOCS3	0.02759
ARHGAP3	0.027598
CCDC33	0.027617
CCNC	0.027627
SOCS1	0.027665
TLR9	0.027678

TAF9B	0.027685
ALDH8A1	0.027733
SNORA74.	0.027773
PCDHGC4	0.027778
RUBCN	0.027818
GPBP1L1	0.027841
ADPGK	0.02786
SGCZ	0.027867
LRRTM3	0.027911
MIR449C	0.027994
PSMD1	0.028059
MIR339	0.028079
NPTX2	0.028091
SLFN13	0.028095
NUP35	0.028135
DMRT1	0.028241
TAF6L	0.02826
MIDN	0.028271
FAM13A-/	0.028288
MIR548F5	0.028304
C3orf20	0.028312
GGPS1	0.028361
MBTPS2	0.028431
USF1	0.028496
CATSPER3	0.028607
C17orf80	0.028708
SECTM1	0.028724
ZBTB1	0.028732
OSM	0.028742
FBXL12	0.028786
SLC17A3	0.028809
HIBCH	0.028816
LOC10050	0.028843
RPSAP52	0.028903
CCDC150	0.028907
CEBPB	0.028924
LCE2D	0.029032
MYOZ3	0.029056
EEA1	0.029072
DTX1	0.029081
TRIM2	0.02911
RIC8A	0.029185
BTBD6	0.029204
NUP88	0.029228
DEK	0.029334
ZFAND2A	0.029337
METRNL	0.029374
ANKMY1	0.029391
SRP54	0.029393
PCDHGA5	0.02942
SRSF1	0.029464
TMEM52B	0.029469
PRDM9	0.029547
OR6S1	0.02955
SAR1B	0.029574
RBM38	0.029575
MFSD9	0.029628
SPRN	0.029631

KIRREL3	0.029696
RBBP4	0.029716
OR10K1	0.029717
SMIM30	0.029744
SRP9	0.029759
MDFIC	0.029831
PLCD4	0.029835
SBF1	0.029846
GORASP2	0.029855
BPIFA2	0.02994
FOSL2	0.030106
RBBP5	0.03029
SHOX	0.030363
KCNMB2	0.030433
LINC0125:	0.030436
MIR16-2	0.030486
LINC0096(	0.030526
RABL3	0.030564
NCOA2	0.030586
PLEKHM2	0.030652
CTSK	0.030706
CCDC83	0.030714
UBN1	0.030733
UBE2DNL	0.030735
C8orf48	0.030742
ARID3B	0.030834
TTLL10	0.030881
CASP8	0.03089
SEC1P	0.03114
ODF3L2	0.031273
SNORA71	0.03141
KMT5C	0.031422
PPIEL	0.03143
EFCAB13	0.031531
UTP11	0.031536
SSX5	0.031569
MORC3	0.031587
CLDN8	0.031603
OR2T3	0.031614
TMUB2	0.031654
PCDHA12	0.031705
VRK1	0.031858
KLHDC1	0.031867
TNK2	0.031912
CEP120	0.03192
PLXNB3	0.031922
FAM90A5I	0.031937
ADAMTS1	0.031943
GALNT13	0.032031
LGALS2	0.032032
LOC64518	0.03213
RBM12	0.03222
SLC25A24	0.032223
TMEM86B	0.032233
CDC42SE1	0.032262
AKAIN1	0.032303
GUCY1B1	0.032386
DARS1	0.032396

SNORD11	0.032403
BABAM2	0.03241
SPATA17	0.032625
CSKMT	0.032673
MICAL2	0.032757
MDM1	0.032786
PCBP3	0.03279
H3-5	0.032802
NUP54	0.032817
PHF20	0.032846
CSTF2	0.032865
ILRUN	0.032867
AP3D1	0.032919
TSPYL6	0.032945
AMTN	0.032971
DISC2	0.033016
GALNT15	0.03308
XPO6	0.033086
TRABD	0.033155
SSBP1	0.033237
EIF4E	0.033254
WWC2	0.033279
SERPINA1	0.033365
CPM	0.033394
ZBTB38	0.033435
MED19	0.033484
CPSF3	0.033541
LRIF1	0.033553
RBP5	0.033638
PCBP1-AS	0.033687
LINC0003	0.0337
MTX2	0.033719
MLLT10	0.033808
CD24	0.033815
RABGEF1	0.033914
SRFBP1	0.033941
GABRA5	0.034009
LEO1	0.03404
SKA2	0.034099
PELI1	0.034178
C16orf72	0.034199
PPP3R1	0.034266
CCDC90B	0.034293
LOC44091	0.034328
NEDD1	0.03434
TSPOAP1	0.034375
OVGP1	0.034443
RNF207	0.034517
ST7-OT3	0.034522
INHBC	0.034523
OR5T3	0.034534
ORC5	0.034568
THNSL2	0.034597
SCRT1	0.034617
NAMPT	0.034621
NCOA3	0.034746
XPNPEP1	0.034774
LCN15	0.034798

TAMM41	0.034806
ACVR2A	0.034818
DLX3	0.034828
SYCP3	0.034829
ZYG11A	0.034905
VPS16	0.034923
H2BU1	0.034989
IL12RB2	0.03499
KIF21B	0.034992
APOA2	0.035095
TTC16	0.035098
SNAP23	0.03512
JAGN1	0.035203
LRR1	0.035326
ADGRE4P	0.035344
KBTBD12	0.035496
LPAR2	0.035549
NPAS3	0.035599
PDHB	0.035602
AP2A2	0.035643
FBXW10	0.035663
SSX3	0.035705
PRXL2C	0.035719
PIK3C2A	0.035957
MRPL3	0.035966
PNPLA6	0.035987
VPS35	0.036015
BATF3	0.036016
UPK1B	0.036033
HSPA8	0.036087
MRPL42	0.036092
UNC119	0.036111
ARMC10	0.036119
GTF2E1	0.03612
APIP	0.03622
EIF2A	0.036275
SCMH1	0.036282
LTB4R2	0.036294
ZNHIT1	0.03633
H4C6	0.036342
MIR548A3	0.036357
CYB5B	0.036401
LOC44124	0.036434
LDB2	0.036465
EFEMP1	0.036563
ZSCAN4	0.036581
C1orf194	0.036601
NKX3-2	0.036702
TGFBR1	0.036713
THOC5	0.036717
SLC13A4	0.036717
ZMYND15	0.036732
DDX52	0.03674
LEAP2	0.036749
NEUROD4	0.036757
ZNF736	0.036759
KCNN2	0.036767
LINC0020(	0.036813

NUDT21	0.036894
STXBP3	0.036904
SNORA5B	0.036922
UEVLD	0.036925
FMO6P	0.036967
LINC0066‹	0.037017
ATP2C1	0.037023
ZC3H12A	0.037032
BBOX1	0.037058
NRIP3	0.037066
INPP5K	0.037076
FAM50A	0.037179
PSMD7	0.037187
YEATS4	0.037217
NOS2	0.037377
RNGTT	0.037445
ITPR2	0.037589
PDCD6IP	0.037619
UBXN6	0.037657
TATDN2	0.037659
CBX4	0.037684
HAAO	0.03772
ADAM20P	0.037739
GML	0.037743
OR4S1	0.03775
MGAT4FP	0.037791
LINC0271‹	0.03787
FOXD4L6	0.037904
TNPO3	0.037924
NAA10	0.037946
COLQ	0.037968
CES2	0.038056
PEX13	0.038118
NCOR1P1	0.038122
FKSG29	0.038147
SLC25A51	0.038171
SGSM1	0.038174
ABHD17B	0.03825
OIP5-AS1	0.038299
DNAJC13	0.038367
COL11A1	0.038402
SNORD32	0.038513
FNDC8	0.038522
ADAM3A	0.03855
EEF1AKM1	0.038589
PEX11B	0.038595
PPA1	0.038617
ENPP2	0.038633
LIMD2	0.038688
RALA	0.03874
MED10	0.038799
HSP90AA1	0.038811
SMARCA5	0.03896
TMEM38B	0.03896
REV3L	0.038997
IL3RA	0.039022
MXD1	0.039052
HNRNPA1	0.039106

ZNF124	0.039166
TSSC4	0.03918
GLP1R	0.039278
PROK2	0.039307
SCN5A	0.039355
CPE	0.039402
HAO2	0.039405
NIF3L1	0.039447
LZIC	0.039556
GOLGA7B	0.039561
BCRP2	0.039586
PLTP	0.039608
EIF4A2	0.039659
CEP170	0.039667
PTCH2	0.039711
DAZ2	0.039714
PPIL1	0.039743
GPHB5	0.039755
SLC5A11	0.03977
RCN3	0.039822
RNF185	0.039851
MIR371A	0.039853
EBF4	0.040028
PREX1	0.040059
ZKSCAN1	0.040088
TBC1D25	0.040112
FCGR2B	0.040133
LOC10013	0.040173
RBM41	0.040181
GGTLC2	0.040197
RALGDS	0.040221
CSNK1G2	0.040234
XKR8	0.040282
RAD21-AS	0.040309
DLEC1	0.04034
YLPM1	0.040385
ROPN1L	0.040443
ELOF1	0.04045
RSPRY1	0.040487
ASB2	0.040493
P2RY12	0.040509
PRKAA2	0.040515
CD200	0.040534
POGLUT3	0.040541
CACNG1	0.040597
OXA1L	0.040617
TSBP1	0.04062
CCER1	0.040634
ME2	0.040668
ADAM10	0.040807
TMED4	0.040849
C1QTNF9	0.040935
PATE3	0.040953
KIF13B	0.04097
CRCT1	0.040979
CPA1	0.041126
GTF2H2	0.041143
CLCN3	0.041194

MGLL	0.041219
LINC0064:	0.041262
KCTD7	0.041347
TSPY2	0.041421
ZNF566	0.041436
BCO1	0.04144
MIR1275	0.041461
GNL2	0.041461
PHYKPL	0.041467
TRMT1L	0.041528
TMEM191	0.041533
NAIF1	0.041628
ZNRF2P1	0.041644
RPL22	0.041669
ATP6V0B	0.04169
COQ10B	0.041691
APEX1	0.041704
CETN2	0.041776
GPR20	0.041785
PCSK1N	0.041849
PRR23A	0.04193
HOXB6	0.042035
ATG2A	0.042088
MIR1225	0.042129
PIANP	0.042153
ADAMTS1	0.042208
CHD2	0.04223
IBTK	0.042291
KIAA1191	0.042302
TMEM134	0.042336
SEL1L2	0.042411
NIT1	0.042428
UBA5	0.04245
LINC0162:	0.042478
C9orf24	0.042539
FIP1L1	0.042583
IER5	0.042584
LOC64338	0.042585
DCAKD	0.042728
CCDC43	0.042738
RAPGEF1	0.042746
PDIA3	0.042853
CALR3	0.042879
TRIM64B	0.042912
KLK12	0.042914
XKR6	0.042946
GPR107	0.042985
PUS7L	0.043081
RTF1	0.04313
MFHAS1	0.043164
ANKDD1A	0.043172
HEPACAM	0.043256
ZNF286A	0.043283
ZFAND3	0.043328
DCAF7	0.043413
MIR1284	0.043539
SGPP1	0.043668
PRG2	0.043775

MIR942	0.043837
ZDHHC18	0.043838
KLK10	0.043902
MIR557	0.043985
DEFB109A	0.04401
FHL1	0.044013
ZDHHC8	0.044016
CYBA	0.044023
COL27A1	0.044127
FBXW2	0.044136
LTBP1	0.04415
CALCB	0.044177
WFDC5	0.044182
MTIF2	0.044199
DBR1	0.044305
SERPINA6	0.044315
RAB24	0.044351
PDZD4	0.044357
PPP6R1	0.044387
GRAMD4	0.044395
TBC1D23	0.044535
ZNF300P1	0.044545
LINC0136	0.044546
RNF31	0.044681
H6PD	0.044728
FOXO3	0.044729
MZT1	0.044747
HCG4	0.044777
TCERG1	0.044897
SCN7A	0.044897
NAP1L5	0.044956
MAP1LC3	0.044966
MYO18B	0.044967
RBAK	0.045143
DAP3	0.045171
SAP30L	0.045173
TMEM99	0.045251
TRMT2A	0.045261
MIR510	0.045317
SNORD11	0.045362
PLCXD2	0.0454
TCHHL1	0.045427
RERGL	0.045453
GYS2	0.045493
CDK19	0.045499
MCL1	0.045563
SLC2A7	0.045566
VASH1	0.045573
ALDH5A1	0.045625
CELF1	0.045629
SLC37A3	0.045693
KIF5B	0.045712
DVL1	0.045712
FZR1	0.045771
EXD2	0.045784
MIR106A	0.0458
SMC1A	0.045867
SOS2	0.045883

MAP3K8	0.045937
PGM3	0.045999
BUB1	0.046058
RASSF7	0.04617
MRPS18B	0.046182
REX1BD	0.04622
ELF2	0.046238
UBA3	0.046405
TSNAX	0.046416
PNLIPRP1	0.046418
FAM166C	0.046424
KHNYN	0.046432
SS18	0.046455
CLNS1A	0.046482
LIN9	0.046483
WDR97	0.046621
INTS1	0.046646
KLRC2	0.046668
SOD2	0.046718
TTTY6	0.046719
KCTD16	0.046728
ADAMTSL	0.046731
RNF11	0.04674
TMEM164	0.046792
ABHD1	0.046794
AGMAT	0.046806
METTL14	0.046816
SEMA6B	0.046821
SLC7A6OS	0.046901
CHRNA2	0.046967
MIR22HG	0.046991
CCDC179	0.046993
BPHL	0.047018
CERS5	0.047041
LINC0163(	0.047061
ITGB3BP	0.04707
SERP2	0.047133
ZNF766	0.047187
SLC7A4	0.047277
ZNF461	0.047279
CDC14C	0.047324
GGT7	0.047328
XYLT1	0.047391
SLC2A2	0.047407
RASSF1	0.047407
NACC2	0.047421
SLC25A34	0.047462
GNPAT	0.047468
KRTAP13-	0.04752
CD200R1	0.047554
POLR2F	0.047662
RPN2	0.047707
MIR564	0.047719
CEP55	0.047728
MARCHF8	0.047845
FBXL15	0.047848
PAIP2	0.047872
LOC64647	0.047877

TFEB	0.047888
ZBED1	0.047922
R3HCC1L	0.048
DCAF16	0.048114
SNORD33	0.048148
SPATA2L	0.048198
MIR423	0.048219
SELENOO	0.048288
RALGAPA:	0.04832
PYM1	0.04837
ARHGAP4	0.048514
LMAN2L	0.048519
ZNF501	0.048529
ZNRF1	0.048614
IFNA14	0.048668
PHF14	0.048716
NACA2	0.048776
SERTAD3	0.048913
CD63	0.048914
ALB	0.048924
GREM1	0.048986
MINK1	0.048999
PSMC2	0.049027
GABRR2	0.049101
SLC39A2	0.049108
EXD1	0.04915
MRPS9	0.049178
CARF	0.049184
GABPB1	0.049207
MYBPC3	0.049253
ANAPC1P	0.049313
PMF1	0.049429
MATK	0.049514
CHMP4B	0.049579
COPB2	0.04965
AMOTL2	0.049702
ECEL1	0.049734
PCNX2	0.04976
MS4A5	0.049781
MLLT3	0.049791
LRBA	0.049813
NSL1	0.049851
VANGL2	0.049873
OR8B2	0.049878
NOLC1	0.049891
FASTK	0.049901
FAM95A	0.049912
C1orf131	0.049915
ARMC9	0.049923
MRPS35	0.049928
EHBP1L1	0.049939
