# Supplementary material for: Identification of the Novel Gene Markers Based on the Gene Profile among Different Severity of Obstructive Sleep Apnea
Source: Comput Math Methods Med. 2022 Oct 4;2022:6517965. doi: 10.1155/2022/6517965 (PMC9554663; doi:10.1155/2022/6517965)
Supplement: Supplementary 3 — Supplementary Table 3. Genes in the overlap of two groups of DEGs. [file 6517965.f3.pdf]

LINC00635
PHGR1
LOC284395
C3orf20
SNORD113-2
EXD1
OR51B5
PROB1
PCSK1N
SNORD114-13
SYCP1
WFDC5
GHRHR
SERPINA12
GPRASP1
AASS
LDB1
CALCB
SNORD119
CRYBA4
PLXNB3
IL12RB2
OR4S1
SVEP1
TMCC3
SAXO2
LCE3A
ADCY4
ADPGK
ASIP
CEL
CUBN
TMEM221
FOXH1
DUSP26
NCAM2
TDRD3
NPSR1
KCTD7
KCTD16
ACE
SUSD5
CIDEC
PIGK
CD209
AKAIN1
EEF1AKMT3
RSPO4
CPE
EXOSC8
STARD7-AS1
OR4A16
AMOTL2
DAZ2
CRCT1
UPK1B
ZNF704
KCNN2

LINC00494
SRD5A3
RPA1
MIR942
TIAM2
GLP1R
TUSC7
OR4N3P
RPL22
NPY1R
CPNE4
PLTP
SMC1A
TMEM215
MIR548A3
DNASE1L3
HEPACAM
FZR1
TLDC2
BPHL
EBF4
MIS18A
CNP
FAM197Y2
FOXA2
NOP56
ZNF766
TUBA3C
MLLT3
LINC00668
