# Supplementary material for: Identification of the Novel Gene Markers Based on the Gene Profile among Different Severity of Obstructive Sleep Apnea
Source: Comput Math Methods Med. 2022 Oct 4;2022:6517965. doi: 10.1155/2022/6517965 (PMC9554663; doi:10.1155/2022/6517965)
Supplement: Supplementary 4 — Supplementary Table 4. Six clusters obtained by soft clustering analysis. [file 6517965.f4.pdf]

ID	Cluster
ZNF766	1
TUSC7	1
TMCC3	1
SMC1A	1
OR4S1	1
OR4N3P	1
LCE3A	1
IL12RB2	1
GPRASP1	1
FZR1	1
CRYBA4	1
CD209	1
AMOTL2	1
TUBA3C	2
RPA1	2
NPY1R	2
LINC0066[illegible]	2
DUSP26	2
CPNE4	2
CPE	2
UPK1B	3
TMEM221	3
TDRD3	3
SRD5A3	3
SNORD11	3
SERPINA1	3
PLXNB3	3
PIGK	3
PHGR1	3
NOP56	3
NCAM2	3
MIS18A	3
MIR942	3
MIR548A3	3
LOC2843[illegible]	3
KCTD7	3
KCNN2	3
EXOSC8	3
EEF1AKM[illegible]	3
ZNF704	4
WFDC5	4
TLDC2	4
SNORD11	4
SAXO2	4
RSPO4	4
PCSK1N	4
OR51B5	4
LINC0063[illegible]	4
LINC0049[illegible]	4
GHRHR	4
FOXH1	4
DNASE1L[illegible]	4
CUBN	4
ASIP	4
ADPGK	4
ADCY4	4
AASS	4

STARD7-A	5
RPL22	5
PROB1	5
KCTD16	5
HEPACAM	5
GLP1R	5
FAM197Y2	5
EXD1	5
CRCT1	5
CEL	5
CALCB	5
C3orf20	5
BPHL	5
ACE	5
TMEM215	6
TIAM2	6
SYCP1	6
SVEP1	6
SUSD5	6
SNORD11	6
PLTP	6
OR4A16	6
NPSR1	6
MLLT3	6
LDB1	6
FOXA2	6
EBF4	6
DAZ2	6
CNP	6
CIDEC	6
AKAIN1	6
